# Surveillance of the Second Wave of COVID-19 in Europe: Longitudinal Trend Analyses

**DOI:** 10.2196/25695

**Published:** 2021-04-28

**Authors:** Lori Post, Kasen Culler, Charles B Moss, Robert L Murphy, Chad J Achenbach, Michael G Ison, Danielle Resnick, Lauren Nadya Singh, Janine White, Michael J Boctor, Sarah B Welch, James Francis Oehmke

**Affiliations:** 1 Buehler Center for Health Policy and Economics Feinberg School of Medicine Northwestern University Chicago, IL United States; 2 Feinberg School of Medicine Northwestern University Chicago, IL United States; 3 Institute of Food and Agricultural Sciences University of Florida Gainsville, FL United States; 4 Institute of Global Health Feinberg School of Medicine Northwestern University Chicago, IL United States; 5 Divison of Infectious Disease Feinberg School of Medicine Northwestern University Chicago, IL United States; 6 International Food Policy Research Institute Washington DC, DC United States

**Keywords:** SARS-CoV-2 surveillance, wave two, second wave, global COVID surveillance, Europe Public Health Surveillance, Europe COVID, Europe surveillance metrics, dynamic panel data, generalized method of the moments, Europe econometrics, Europe SARS-CoV-2, Europe COVID surveillance system, European COVID transmission speed, European COVID transmission acceleration, COVID transmission deceleration, COVID transmission jerk, COVID 7-day lag, SARS-CoV-2, Arellano-Bond estimator, GMM, Albania, Andorra, Austria, Belarus, Belgium, Bosnia and Herzegovina, Bulgaria, Croatia, Czech Republic, Denmark, Estonia, Finland, France, Germany, Greece, Greenland, Hungary, Iceland, Ireland, Isle of Man, Italy, Latvia, Liechtenstein, Lithuania, Luxembourg, Moldova, Monaco, Montenegro, Netherlands, Norway, Poland, Portugal, Romania, San Marino, Serbia, Slovakia, Slovenia, Spain, Sweden, Switzerland, Ukraine, United Kingdom, Vatican City

## Abstract

**Background:**

The COVID-19 pandemic has severely impacted Europe, resulting in a high caseload and deaths that varied by country. The second wave of the COVID-19 pandemic has breached the borders of Europe. Public health surveillance is necessary to inform policy and guide leaders.

**Objective:**

This study aimed to provide advanced surveillance metrics for COVID-19 transmission that account for weekly shifts in the pandemic, speed, acceleration, jerk, and persistence, to better understand countries at risk for explosive growth and those that are managing the pandemic effectively.

**Methods:**

We performed a longitudinal trend analysis and extracted 62 days of COVID-19 data from public health registries. We used an empirical difference equation to measure the daily number of cases in Europe as a function of the prior number of cases, the level of testing, and weekly shift variables based on a dynamic panel model that was estimated using the generalized method of moments approach by implementing the Arellano-Bond estimator in R.

**Results:**

New COVID-19 cases slightly decreased from 158,741 (week 1, January 4-10, 2021) to 152,064 (week 2, January 11-17, 2021), and cumulative cases increased from 22,507,271 (week 1) to 23,890,761 (week 2), with a weekly increase of 1,383,490 between January 10 and January 17. France, Germany, Italy, Spain, and the United Kingdom had the largest 7-day moving averages for new cases during week 1. During week 2, the 7-day moving average for France and Spain increased. From week 1 to week 2, the speed decreased (37.72 to 33.02 per 100,000), acceleration decreased (0.39 to –0.16 per 100,000), and jerk increased (–1.30 to 1.37 per 100,000).

**Conclusions:**

The United Kingdom, Spain, and Portugal, in particular, are at risk for a rapid expansion in COVID-19 transmission. An examination of the European region suggests that there was a decrease in the COVID-19 caseload between January 4 and January 17, 2021. Unfortunately, the rates of jerk, which were negative for Europe at the beginning of the month, reversed course and became positive, despite decreases in speed and acceleration. Finally, the 7-day persistence rate was higher during week 2 than during week 1. These measures indicate that the second wave of the pandemic may be subsiding, but some countries remain at risk for new outbreaks and increased transmission in the absence of rapid policy responses.

## Introduction

### Background

**Figure 1:**
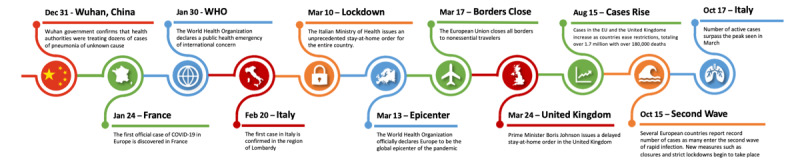
Timeline of COVID-19–related events and decisions made (2020). EU: European Union; WHO: World Health Organization.

The first European COVID-19 case was reported on January 24, 2020, in France, with subsequent cases confirmed in Germany and Finland days later [1]. On March 11, 2020, the World Health Organization (WHO) declared that the spread of the novel coronavirus had exceeded the threshold of a pandemic [2] and, on March 13, 2020, the WHO declared Europe as the global epicenter, when their caseload and deaths exceeded the combined caseload in the rest of the world [1] (See Figure 1). The European Union (EU) closed all external borders on March 17, 2020 [1]. Although the EU coordinated the COVID-19 response between member countries, individual governments enacted separate national policies and made individual decisions regarding border closure and quarantine measures [3]. COVID-19 caseloads decreased for most European countries after peaking in April and May [4].

At present, European countries are experiencing a second wave of COVID-19 [5-11]. The WHO has warned that the death counts in Europe could surpass the peak observed in April 2020 [12]. Nations worldwide are struggling to control COVID-19 transmission by imposing social isolation and economic restrictions, with leaders reluctant to shut down businesses and quarantine citizens again [13,14]. As of February 9, 2021, the WHO reported 106,125,682 confirmed COVID-19 cases and 2,320,497 deaths worldwide [15]. Collectively, 33,534,153 COVID-19 cases have been reported in the EU and the United Kingdom, which have resulted in 740,733 deaths [4].

The World Bank (WB), a global partnership dedicated to reducing poverty and increasing sustainable prosperity in developing nations, divides the world into regions based on shared geographical, development, and cultural or historical features [16]. The Global SARS-CoV-2 Surveillance Project: Policy, Persistence, & Transmission provides surveillance data [17] based on these WB-defined regions. The focus of this study is on the spread of COVID-19 specifically within the Western European region, including Albania, Andorra, Austria, Belarus, Belgium, Bosnia and Herzegovina, Bulgaria, Croatia, Czech Republic, Denmark, Estonia, Finland, France, Germany, Greece, Greenland, Hungary, Iceland, Ireland, Isle of Man, Italy, Latvia, Liechtenstein, Lithuania, Luxembourg, Moldova, Monaco, Montenegro, the Netherlands, Norway, Poland, Portugal, Romania, San Marino, Serbia, Slovakia, Slovenia, Spain, Sweden, Switzerland, Ukraine, the United Kingdom, and Vatican City.

### Outbreak and Governance

#### Policies and Culture

Analysis of COVID-19 cumulative incidence indicates that the drastic measures undertaken by the Italian government slowed the spread of the disease to lower than the expected 7-10 days after restrictions were implemented [18]. The rapid transmission was likely due to high population density [19], and the high case-fatality rate is associated with the older age distribution in Italy, wherein approximately 23% of the Italian population was aged 65 years or older in 2019 [20].

Other factors that influenced the severity of the COVID-19 were family structures, which likely increased interaction among family members [21]. Additionally, Southern European countries engage in physical greetings, with kisses on the cheek and friendly hugs being common in Italy, Spain, and France [22]. These cultural practices may be a contributing factor to the increased transmission of COVID-19 and related mortality in the Southern European countries listed above, where the virus spread very rapidly and yielded severe adverse effects [23].

In contrast, in Northern European countries such as Sweden, children tend to leave home earlier and frequently move farther away from their parents, often to pursue higher education. A “post-nuclear family structure” has developed more rapidly, and children in these countries may have less frequent contact with their families from an earlier age than those in the more traditional Southern European countries [21]. Additionally, personal space is valued to a higher degree, and kissing is less commonly used as a greeting compared to shaking hands or other less physical forms of greeting [24]. Sweden enacted less strict policies than Southern European countries did and saw similar results as countries that enacted late-onset stringent mandates [25]. It is worth noting that Sweden’s per capita COVID-19 death rate far outpaces that of its Scandinavian neighbors, decreasing confidence in their mitigation strategies [26]. At the other extreme lie countries such as Hungary, where the Prime Minister pushed through legislation that allowed him to rule by decree for however long the pandemic continues and mandates jail time for the spread of disinformation, leading to concerns about restrictions on human rights and media freedoms [27].

The United Kingdom, physically and organizationally separated from its European neighbors since leaving the European Union, took a delayed and somewhat hesitant approach to controlling the spread of the virus. The first two COVID-19 cases in the nation were confirmed on January 31, 2020. The Department of Health and Social Care’s coronavirus action plan was approved on March 3, 2020, outlining the country’s plan to deploy four phased actions to deal with the pandemic: Contain, Delay, Research, and Mitigate [28]. The government moved from the Contain phase to the Delay phase on March 12, 2020, after Italy had already locked down, and emphasized testing in hospital settings and not communities, with unrestricted entry to the country via ports and airports [28]. On March 19, 2020, COVID-19 was reclassified from level 4 to a milder threat level (ie, level 3) by the Advisory Committee on Dangerous Pathogens, allowing hospital infection control requirements to be lowered [28]. Finally, on March 24, 2020, the Prime Minister declared an enforceable lockdown across the nation [28], but COVID-19 spread rapidly throughout the United Kingdom, leading Europe in COVID-19–related deaths at over 43,579 [4]. Many European countries are experiencing a second wave of infections, with surging daily case numbers in France, Spain, the Netherlands, and the United Kingdom, and the WHO warning that, within the coming months, daily death counts could surpass the April peak observed in Europe [12]. National governments are struggling to control the infection due to increased pushback from local governments who are reluctant to shut down businesses and quarantine citizens a second time after being allowed to open up [13].

#### Economics and Food Insecurity

An important impact of the pandemic is the risk of food insecurity in vulnerable nations such as Ukraine and Moldova [29]. Much of the population in Ukraine lacks the ability to buy a sufficient amount of healthy food and often resides in conflict-affected areas of the country. Moreover, the current pandemic threatens to impact Ukraine’s wheat exportation and livestock processing, which could create even more scarcity in affordable food for its citizens [30,31]. Ukraine responded with early restrictive policies in response to widespread fear among citizens, and the country ended up reporting fewer cases than Russia and Belarus, indicating that its response was most likely effective in slowing disease transmission [32]. However, economic growth in Ukraine was stable at 3.2% in 2019, but the pandemic has forced a sudden slowdown in economic activity; the future of the economy will be dependent on the country’s ability to support investment and diversify exports after the pandemic subsides [33].

Economic growth in Moldova had already declined sharply to 0.2% in late 2019, and the unemployment rate saw an increase compared to 2018 [33]. Many citizens of Moldova rely heavily on food self-provisioning or food sharing within village networks [34]. Poverty is expected to increase in response to the COVID-19 pandemic, and the effects will likely impact households with inadequate insurance mechanisms. The maintenance of food security and economic stability will depend on the government’s ability to alleviate food shortages and compensate for lost income, as well as to support jobs and growth when the crisis subsides [33].

### Surveillance

Public health surveillance informs policy on “flattening the curve” of COVID-19 spread [17,35-37]. Epidemiologists have utilized various modeling techniques to forecast COVID-19 case numbers and attributed deaths [38-42]. The European Center for Disease Control, the WHO, and the Center for Systems Science and Engineering at Johns Hopkins University have developed tracking tools [11,38]. Although helpful, these static metrics are limited by incomplete case ascertainment and data contamination [17,36]. Existing surveillance is a proxy for the true COVID-19 caseload because public health surveillance systems tend to pick up the most severe cases [43,44], which is especially problematic when tracking SARS-CoV-2 infections because most carriers are asymptomatic or presymptomatic or may have mild symptoms [45-48]. Therefore, public health surveillance that can control for these limitations are needed. Moreover, metrics that detect the speed of transmission of the novel coronavirus, shifts in the pandemic, and acceleration of the speed and persistence of COVID-19 based on prior infections are needed to supplement existing measures.

### Significance

Ideally, the development of a more advanced methodology for tracking and estimating COVID-19 transmission in regions within Europe will allow for a more reliable analysis of which policies are effective and what other factors may be associated with transmission rates. Public health departments, in addition to several universities and media outlets, are tracking COVID-19 metrics by using raw data, including the number of new cases, diagnostic tests, positive results, transmission rates and deaths, in addition to other measures such as local hospital capacity [4,49-57]. To remove temporal effects, many surveillance systems have shifted to 7-day moving averages to counter the dearth of reporting during holidays and weekends. Although moving averages temper volatility of data and testing or reporting affects, surveillance is still limited by missing cases. General public health surveillance is helpful and provides a proxy of the pandemic, but surveillance data are still limited by significant bias due to undercounts, reporting delays, testing errors, dearth of testing, asymptomatic carriers, and other types of data contamination. In fact, surveillance systems are predicated on the fact that they tend to include only the more severe cases, whereas mild cases and undiagnosed infections and deaths are excluded [43,44].

To that end, the objective of our study is to use a longitudinal trend analysis study design in concert with *Dynamic Panel Modeling* and *Method of Moments* to correct for existing surveillance data limitations [17,36]. Specifically, we will measure significant weekly shifts in the increase, decrease, or plateaued transmission of COVID-19. We will also measure the underlying causal effect from the previous week that persists through the current week, with a 7-day persistence rate to explain a clustering-declustering effect. The 7-day persistence represents an underlying disease transmission wave, wherein a large number of transmissions 7 days ago that resulted in a large number of infections today then *echoes* forward into a large number of new transmissions and, hence, a large number of new cases 7 days forward. An example of the 7-day lag would be large sporting events in the United Kingdom that drew huge crowds weekend after weekend even after new COVID-19 cases were confirmed in the country. Other potential “superspreader” events such as the exportation of COVID-19 cases from a popular ski town in the Austrian Alps back in March 2020 [58], would certainly contribute to this persistence effect as well. In summary, we will measure negative and positive shifts in the transmission of SARS-CoV-2 or the acceleration or deceleration rates. Our surveillance metric will provide public health surveillance data to inform governments in decision-making regarding disease control, mitigation strategies, and reopening policies as they continue to manage this unprecedented situation.

## Methods

Our World in Data [59] compiles data from multiple sources on the web. Data for the most recent 7 weeks were accessed from the GitHub repository [60]. This resulted in a panel of 39 countries in Western Europe with 62 days in each panel (n=2418). Based on published reports [16,61], an empirical difference equation was specified in which the number of new positive cases in each country at each day is a function of the prior number of cases, the level of testing, and weekly shift variables that measure whether the contagion was growing faster, at the same speed, or slower than in the previous weeks. This resulted in a dynamic panel model that was estimated using the generalized method of moments (GMM) approach by implementing the Arellano-Bond estimator in R [17,36].

## Results

### Country Regression Results

Regression results are presented for 39 European countries in Table 1. Weekly surveillance data presented in Tables 2-6 are based in part on these regressions. Data for 44 European countries were collected, but data for 5 countries were excluded in the regression analysis due to missing data. The regression Wald statistic is significant (*X^2^*_8_=4980; *P*<.001). The Sargan test was not significant, failing to reject the validity of overidentifying restrictions (*X^2^*_511_=39; *P=*.39).

The coefficient for the 7-day lag was positive and statistically significant (0.90, *P*<.001), indicating the number of infections 7 days prior to the study had a positive relationship that echoed forward 7 days later. The shift parameter 14 days ago was negative and statistically significant (coefficient –0.30, *P*<.001), suggesting that exogenous shift events had a negative effect on total case numbers (Table 1).

**Table 1:** Arellano-Bond dynamic panel data model of COVID-19 dynamics at the country level in Europe.

Variable	Coefficient	*P* value
7-day lag	0.90	<.001
Cumulative tests	–0.000	.42
7-day lag shift	–0.30	<.001
Weekend	–2.1	.02

**Table 2:** Static surveillance metrics for European countries for the week of January 4-10, 2021.

Country	New weekly COVID-19 cases	Cumulative COVID-19 cases	7-day moving average of new COVID-19 cases	Infection rate per 100,000 population	New weekly deaths	Cumulative deaths due to COVID-19	7-day moving average of new COVID-19–related deaths	Deaths rate per 100,000 population
Albania	562	63,595	593.86	19.53	8	1241	6.86	0.28
Andorra	0	8586	56.29	0.00	0	85	0.14	0
Austria	1651	380,722	2136.29	18.33	36	6723	57	0.40
Belarus	1833	212,201	1748.43	19.40	10	1517	9.43	0.11
Belgium	1569	664,263	2036	13.54	40	20,078	53.86	0.35
Bosnia & Herzegovina	254	115,633	426.86	7.74	25	4330	28.43	0.76
Bulgaria	105	208,511	780	1.51	29	8126	64	0.42
Croatia	646	219,993	1005	15.74	26	4368	42.29	0.63
Czech Republic	8449	831,165	12,954.86	78.90	137	13,115	165	1.28
Denmark	1246	182,161	1829	21.51	28	1571	28.14	0.48
Estonia	427	33,516	626.43	32.19	5	283	5.57	0.38
Finland	198	38,590	259.71	3.57	0	586	3.57	0
France	159,44	2,840,864	18,269.86	24.43	151	67,885	388.71	0.23
Germany	948	1,929,410	20,787.71	1.13	339	40,936	877.86	0.40
Greece	445	144,738	662.71	4.27	36	5263	43.71	0.35
Hungary	1778	342,237	2034.57	18.41	94	10,648	109.14	0.97
Iceland	10	5890	19.43	2.93	0	35	0	0
Ireland	6886	147,613	6532.29	139.46	8	2344	12.14	0.16
Italy	18,625	2,276,491	17,292.14	30.80	361	78,755	489.00	0.60
Latvia	616	49,568	1010.14	32.66	31	849	24.14	1.64
Lithuania	1492	159,672	1862.14	54.81	26	2197	79.14	0.96
Luxembourg	0,	47,744	189.86	0.00	0	527	4.57	0.00
Malta	184	14,396	187.71	41.67	1	233	1.86	0.23
Moldova	298	149,391	502.57	7.39	9	3139	14.57	0.22
Netherlands	6655	885,098	7485.14	38.84	55	12,461	107.71	0.32
Norway	555	55,474	679.71	10.24	0	472	5.14	0
Poland	9133	1,385,522	9565.71	24.13	178	31,189	295.71	0.47
Portugal	7502	483,689	8062.14	73.57	102	7803	97.86	1.00
Romania	3082	671,284	4407.86	16.02	62	16,654	96.43	0.32
San Marino	0	2628	28.57	0.00	0	64	0.71	0
Serbia	3564	359,689	2259.86	40.79	69	3582	36.71	0.79
Slovakia	2973	208,209	2963.71	54.45	82	2919	85.86	1.50
Slovenia	763	139,281	2027.86	36.70	25	2998	27.86	1.20
Spain	0	2,050,360	17,442.14	0	0	51,874	148.14	0
Sweden	0	489,471	7441.71	0	0	9433	100.86	0.00
Switzerland	0	477,983	3669.57	0	14	8267	74.29	0.16
Ukraine	5322	1,150,265	6161.14	12.17	115	20,641	144.43	0.26
United Kingdom	55,026	3,081,368	59,809.86	81.06	567	81,567	918.57	0.84
Europe	158,741	22,507,271	208,581.43	26.52	2669	524,758	4649.43	0.45

**Table 3:** Static surveillance metrics for European countries for the week of January 11-17, 2021.

Country	New weekly COVID-19 cases		Cumulative COVID-19 cases	7-day moving average of new COVID-19 cases	Infection rate per 100,000 population	New weekly deaths	Cumulative deaths due to COVID-10	7-day moving average of new COVID-19–related deaths	Deaths rate per 100,000 population
Albania	474	67,690		585	16.47	7	1277	5.14	0.24
Andorra	45	9083		71	58.24	0	91	0.86	0
Austria	1267	393,778		1865.14	14.07	29	7082	51.29	0.32
Belarus	1924	22,5461		1894.29	20.36	9	1582	9.29	0.10
Belgium	1630	678,839		2082.29	14.06	39	20,435	51	0.34
Bosnia & Herzegovina	0	11,7011		196.86	0	0	4411	11.57	0
Bulgaria	77	211,813		471.71	1.11	9	8483	51	0.13
Croatia	379	224,954		708.71	9.23	28	4616	35.43	0.68
Czech Republic	5253	889,159		8284.86	49.05	123	14,338	174.71	1.15
Denmark	889	189,767		1086.57	15.35	28	1776	29.29	0.48
Estonia	388	37,079		509	29.25	5	325	6	0.38
Finland	236	40,337		249.57	4.26	0	618	4.57	0
France	37,405	2,969,091		18,318.14	57.31	329	70,422	362.43	0.50
Germany	11,484	2,050,129		17,245.57	13.71	437	46,901	852.14	0.52
Greece	237	148,607		552.71	2.27	28	5469	29.43	0.27
Hungary	1241	351,828		1370.14	12.85	77	11,341	99	0.80
Iceland	0	5956		9.43	0	0	35	0	0
Ireland	2946	172,726		3587.57	59.66	13	2608	37.71	0.26
Italy	125,44	2,381,277		14,969.43	20.75	377	82,177	488.86	0.62
Latvia	567	55,664		870.86	30.06	17	978	18.43	0.90
Lithuania	836	167,516		1120.57	30.71	31	2445	35.43	1.14
Luxembourg	0	48,630		126.57	0	0	549	3.14	0
Malta	141	15,588		170.29	31.93	1	239	0.86	0.23
Moldova	214	152,854		494.71	5.30	5	3250	15.86	0.12
Netherlands	5643	925,355		5751	32.93	41	13,107	92.29	0.24
Norway	206	58,651		453.86	3.80	0	517	6.43	0.00
Poland	5970	1,435,582		7151.43	15.77	142	33,355	309.43	0.38
Portugal	10,385	549,801		9444.57	101.85	152	8861	151.14	1.49
Romania	2156	693,644		3194.29	11.21	57	17,221	81	0.30
San Marino	0	2778		21.43	0	0	65	0.14	0
Serbia	1317	372,533		1834.86	15.07	20	3750	24	0.23
Slovakia	573	223,325		2159.43	10.50	57	3475	79.43	1.04
Slovenia	569	149,125		1406.29	27.37	40	3180	26	1.92
Spain	0	2,252,164		28,829.14	0	0	53,314	205.71	0
Sweden	0	523,486		4859.29	0	0	10,323	127.14	0
Switzerland	0	495,228		2463.57	0	7	8682	59.29	0.08
Ukraine	6398	1,198,512		6892.43	14.63	130	21,767	160.86	0.30
United Kingdom	38,670	3,405,740		46,338.86	56.96	682	89,429	1123.14	1
Europe	152,064	23,890,761		17,7288	25.40	2920	558,494	4819.43	0.49

**Table 4:** Novel surveillance metrics for European countries for the week of January 4-10, 2021.

Country	Speed^a^ (weekly average of new daily cases per 100,000 population)	Acceleration^b^ (weekly average, per 100,000 population)	Jerk^c^ (per 100,000 population)	7-day persistence effect on speed (number of new daily cases per 100,000 population attributed to new cases 7 days ago)
Albania	20.64	0.57	0.67	9.48
Andorra	72.85	–4.81	–13.68	41.13
Austria	23.72	0.29	–1.11	13.20
Belarus	18.50	–604,7296.00	0.20	11.98
Belgium	17.57	0.90	–0.47	8.23
Bosnia & Herzegovina	13.01	–1.08	–3.08	7.71
Bulgaria	11.23	–0.14	–0.25	6.58
Croatia	24.48	–0.17	–4.16	16.75
Czech Republic	120.97	4.58	–8.31	55.90
Denmark	31.58	0.17	–0.15	22.78
Estonia	47.22	0.92	–0.90	24.14
Finland	4.69	0.08	–0.24	2.78
France	27.99	0.75	–2.90	12.66
Germany	24.81	–1.60	-3.64	12.81
Greece	6.36	0.08	–0.66	3.82
Hungary	21.06	0.70	–1.23	10.58
Iceland	5.69	0.42	–0.13	1.78
Ireland	132.29	5.57	1.38	27.34
Italy	28.60	1.03	–0.89	15.27
Latvia	53.55	0.36	–6.12	28.01
Lithuania	68.40	1.33	0.57	50.47
Luxembourg	30.33	0	0	16.50
Malta	42.51	3.20	–1.88	14.69
Moldova	12.46	0.42	0.46	9.59
Netherlands	43.68	–0.67	0.38	29.34
Norway	12.54	0.28	0.33	5.78
Poland	25.27	1.26	–0.15	13.75
Portugal	79.07	5.77	–2.97	27.46
Romania	22.91	0.04	–2.35	10.96
San Marino	84.20	0	0	49.73
Serbia	25.86	2.61	5.73	17.46
Slovakia	54.28	4.59	–1.52	31.29
Slovenia	97.54	0.14	-10.14	42.36
Spain	37.31	0	–203.00	13.43
Sweden	73.69	0	0	35.06
Switzerland	42.40	0	0	23.85
Ukraine	14.09	0.14	0.23	9.96
United Kingdom	88.10	–0.03	–0.50	46.37
Region	37.72	0.39	–1.30	18.97

^a^Speed: Daily positive cases per 100,000 population.

^b^Acceleration: day-to-day change in the number of positive cases per day.

^c^Jerk: week-over-week change in acceleration.

**Table 5:** Novel surveillance metrics for the week of January 11-17, 2021.

Country	Speed^a^ (weekly average of new daily cases per 100,000 population)	Acceleration^b^ (weekly average per 100,000 population)	Jerk^c^ (per 100,000 population)	7-day persistence effect on speed (number of new daily cases per 100,000 population attributed to new cases 7 days ago)
Albania	20.33	–0.44	–0.07	12.37
Andorra	91.89	8.32	9.24	43.68
Austria	20.71	–0.61	0.27	14.22
Belarus	20.05	0.14	–0.11	11.10
Belgium	17.97	0.08	–0.08	10.53
Bosnia & Herzegovina	6	–1.11	0.89	7.80
Bulgaria	6.79	–0.06	0.27	6.73
Croatia	17.26	–0.93	1.29	14.68
Czech Republic	77.36	–4.26	0.82	72.54
Denmark	18.76	–0.88	1.25	18.93
Estonia	38.37	–0.42	–0.88	28.32
Finland	4.50	0.10	0.44	2.81
France	28.06	4.70	9.05	16.78
Germany	20.58	1.80	3.16	14.88
Greece	5.30	–0.29	0.11	3.81
Hungary	14.18	–0.79	1.10	12.63
Iceland	2.76	–0.42	0.13	3.41
Ireland	72.66	–11.40	–6.74	79.33
Italy	24.76	–1.44	–0.57	17.15
Latvia	46.17	–0.37	0.68	32.11
Lithuania	41.16	-3.44	–0.71	41.02
Luxembourg	20.22	0	0	18.19
Malta	38.57	–1.39	0.87	25.49
Moldova	12.26	–0.30	–0.13	7.47
Netherlands	33.56	–0.84	0.87	26.19
Norway	8.37	–0.92	–0.39	7.52
Poland	18.90	–1.19	0.11	15.16
Portugal	92.62	4.04	1.98	47.41
Romania	16.60	–0.69	0.19	13.74
San Marino	63.15	0	–4.63	50.49
Serbia	21	–3.67	–5.95	15.51
Slovakia	39.55	–6.28	–0.98	32.55
Slovenia	67.64	–1.33	1.07	58.49
Spain	61.66	0	0	22.37
Sweden	48.12	0	0	44.18
Switzerland	28.47	0	0	25.42
Ukraine	15.76	0.35	–0.63	8.45
United Kingdom	68.26	–3.44	0.49	52.83
Europe	33.02	–0.16	1.37	22.62

^a^Speed: Daily positive cases per 100,000 population.

^b^Acceleration: day-to-day change in the number of positive cases per day.

^c^Jerk: week-over-week change in acceleration.

**Table 6:** Difference in 7-day persistence between the two study weeks.

Rank	Week 1 (January 4-10, 2021)			Week 2 (January 11-17, 2021)	
	Country	Difference	Country		Difference
1	Czech Republic	55.90	Ireland		79.33
2	Lithuania	50.47	Czech Republic		72.54
3	San Marino	49.73	Slovenia		58.49
4	United Kingdom	46.36	United Kingdom		52.83

### Interpretation: Europe Regression Results

The lagging indicators and shift parameters suggested recent changes in disease transmission in Europe between November 30, 2020, and January 17, 2021. The shift in the most recent 14 days, or 2 weeks, was negative and statistically significant *(P*<.001). The coefficient for *weekend* was negative and statistically significant (–2.1, *P*<.02), as shown in Table 1.

### Surveillance Results

Tables 2-6 display static and novel dynamic surveillance measures for the weeks of January 4-10, 2021, and January 11-17, 2021. Information pertaining to the prior weeks can be found in Tables S1-S8 of Multimedia Appendix 1. Static measures include the number of new cases during the first day of a given week, cumulative cases, the 7-day moving average of new cases, rate of infection, new deaths during the first day of a given week, cumulative deaths, the 7-day moving average of new deaths, and the rate of deaths (see Tables 2 and 3). The dynamic measures include a temporal element to better understand how past cases affect the present ones and how present cases affect the future ones. Dynamic measures (see Tables 4 and 5) include (1) speed—the number of new observed COVID-19 cases per day per 100,000, averaged over a week; (2) acceleration—the change in speed from the prior week to the current week; (3) jerk—the week-over-week change in acceleration as a function of time over the course of 2 weeks between January 4 and 17, 2021; and (4) the 7-day persistence effect on speed—the average of the number of new cases per day in a given week that are statistically attributable to new cases reported 7 days earlier.

Static measures in Europe for the week of January 4-10, 2021, are presented in Table 2 and those for the week of January 11-17, 2021, are presented in Table 3. New European cases slightly decreased from 158,741 to 152,064 during the first day of each week, with only cumulative cases increasing from 22,507,271 to 23,890,761, which is a weekly increase of 1,383,490 from January 10 to January 17, 2021. Cumulative deaths due to COVID-19 in Europe reached 558,494 by January 17, 2021. The 7-day moving average of new cases totaled 208,581 in the first week and 177,288 in the second week.

France, Germany, Italy, Spain, and the United Kingdom had the largest 7-day moving averages for new cases of infection at 18,269, 20,787, 17,292, 17,442, and 59,809 during the week of January 4-10, 2021. In the second week (January 11-17, 2021), 7-day moving averages increased to 18,318 and 28,829 for France and Spain, respectively. The 7-day moving average for Germany, Italy, and the United Kingdom decreased to 17,245, 14,969, and 46,338, respectively. The infection rate per 100,000 people during the week of January 4-10, 2021, was the highest in Ireland and the United Kingdom at 139.5 and 81.06, respectively. The Czech Republic, Portugal, and Lithuania reported the next highest rates at 78.90, 73.59, and 54.81 per 100,000 population. These 5 countries with the highest infection rates reported a change for the week of January 11-17, 2021, thereby also changing the ranking and magnitude of rates. The top 5 countries by infection rate in week 2 were Portugal at 101.85, Ireland at 59.66, Andorra at 58.24, France at 57.31, and the United Kingdom at 56.94 per 100,000 population.

During the week of January 4-10, 2021, the highest death rates were reported in Latvia, Slovakia, and the Czech Republic at 1.64, 1.50, and 1.28 per 100,000 population. The following week, the European countries with the highest death rates were Slovenia, Portugal, and the Czech Republic at 1.92, 1.49 and 1.15 per 100,000 population.

**Figure 2:**
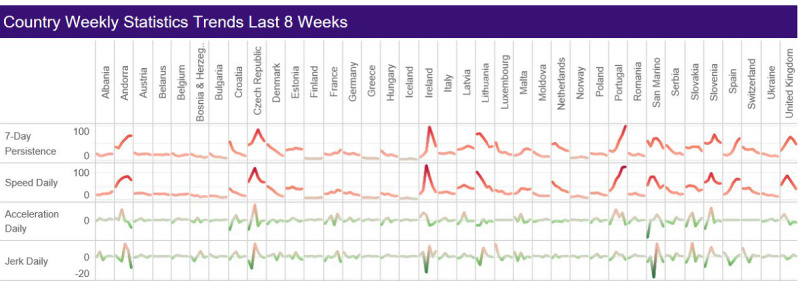
Weekly SARS-CoV-2 trends by country in Europe (December 7, 2020, to January 17, 2021; data source: [62]).

Tables 4 and 5 and Figure 2 (data sourced from The Global SARS-CoV-2 Surveillance Project [62]) display the dynamic metrics that offer a more temporal view of these data. Novel metrics are also displayed in Multimedia Appendices 2-4. From the week of January 4-10 to the week of January 11-17, 2021, in Europe, the dynamic measures of speed decreased (37.72 to 33.02 per 100,000), acceleration decreased (0.39 to –0.16 per 100,000), and jerk increased (–1.30 to 1.37 per 100,000). Speed was the highest and decreasing in Ireland (132.29 to 72.66 per 100,000), the Czech Republic (120.97 to 77.36 per 100,000), and Slovenia (97.54 to 67.64 per 100,000) during both weeks. Acceleration was the highest in Portugal, Ireland, and Slovakia in the week of January 4-10, 2021, at 5.77, 5.57, and 4.59 per 100,000 population, respectively. Only Ireland had a positive jerk during this time. Andorra, France, and Portugal had the largest acceleration rates during the week of January 4-10, with reported increases to 8.32, 4.70, and 4.04 per 100,000 population, respectively. Jerk was the highest in Serbia, Ireland, and Albania during the week of January 4-10, 2021, at 5.73, 1.38, and 0.67 per 100,000 population. Andorra, France, and Germany reported the highest jerk rates per 100,000 in the week January 11-17, 2021, at 9.24, 9.05, and 3.16, respectively.

The 7-day persistence difference in Table 6 demonstrates the changes in 7-day persistence for the top 4 European countries between January 4-10 and January 11-17, 2021, suggesting an underlying shift that significantly increased persistence for some countries but significantly decreased persistence for other countries during the week of January 11-17. Only two of the countries were in the top 4 for both weeks (ie, the Czech Republic and United Kingdom). Lithuania and San Marino were in the top 4 for the first week, but Lithuania decreased to 41.01 in the second week. San Marino slightly increased from 49.73 to 50.49, but did not make it into the top 4 for the second week.

Among the top 5 countries by population (Table 7), Germany and France remained relatively stable, and the United Kingdom had the highest indicators of cause for alarm, with positive increases in speed and persistence but slightly negative decreases in acceleration and jerk. Smaller countries such as the Czech Republic, Ireland, Andorra, and Portugal reported higher positive increases in speed, acceleration, jerk, and persistence.

**Table 7:** Most populous European countries.

Country	Population as of 2020
Germany	83,783,942
United Kingdom	67,886,011
France	65,273,511
Italy	60,461,826
Spain	46,754,778

## Discussion

### Principal Results

Thus far, European COVID-19 infection surveillance has depended on static metrics with limited insight into longitudinal pandemic progression. Dynamic metrics provide an additional lens for surveillance that better captures the evolving prevalence of disease. After combining static and dynamic metrics, some European countries stand out as with the highest risk for uncontrolled growth. These high-risk countries must maintain transmission mitigating policies if they are to protect their citizens and the citizens of neighboring countries.

Europe, as a region, is still experiencing high COVID-19 case rates, but these appear to be trending downward as the region emerges from its second wave. The 7-day moving average of new cases showed a substantial decrease from 208,581 to 177,288 between January 4 and January 17, 2021. The 7-day moving average of COVID-19–related deaths, however, increased from the week of January 4-10 to the week of January 11-17, 2021. Speed of transmission in the region decreased and acceleration shifted from positive to negative from week 1 to week 2, suggesting that case rates may continue to trend downward in coming weeks. This shift in acceleration implies that the speed was increasing at the beginning of the study period but entered a downward trajectory by the end. However, jerk shifted from negative to positive during these two weeks, indicating that the downward trend in acceleration was slowing toward the end of the study period. Interventions and continued precautions will be necessary to maintain a decreasing 7-day moving average of new cases and to continue the downward trajectory of speed and acceleration.

Infection rates show the countries that were the hardest hit at the time of data collection. The top 5 most populous countries in Europe are Germany, the United Kingdom, France, Italy, and Spain. Unsurprisingly, these 5 countries also had the largest 7-day moving averages for new infections during the study period. The United Kingdom had the second highest infection rate per 100,000 people during week 1 of the data collection, along with the largest 7-day moving average of new cases. This finding indicates that the United Kingdom may be at risk of increasing transmission, but the infection rate per 100,000 people decreased from 81.06 during week 1 to 56.94 in week 2, which is reassuring. Both the speed of virus transmission and acceleration in the United Kingdom decreased over the recorded period as well, but jerk actually increased from –0.49 to 0.50, and the country’s 7-day persistence was the fourth highest in Europe during both weeks, indicating that the United Kingdom does need to stay vigilant and ensure proper enforcement of policies to reduce transmission in order to avoid another outbreak.

France and Germany both reported increases in acceleration over the two weeks, and the jerk transitioned from a negative value to a positive value, putting both countries at risk of experiencing increased growth in the coming weeks. Additionally, Spain had increasing speed and jerk, and its 7-day persistence effect increased over the 2-week period, indicating an increase in forward echoes of COVID-19 cases present in the country. Fortunately, Italy’s speed and acceleration both decreased over the recorded period, and the jerk was negative during both weeks, implying that mitigation strategies are currently proving to be effective in Italy—the country that was initially one of the hardest hit. These 5 most populous countries are responsible for a very significant portion of the total cases in the European region, and they will likely require regional policy coordination for optimal control of virus transmission.

Some smaller countries in the region have also demonstrated dynamic metrics that warrant concern, such as Andorra. The speed increased from week 1 to week 2, and jerk and acceleration both dramatically increased from negative to positive values, indicating that more intense restrictions are likely necessary to slow the spread. Ireland had the highest infection rate per 100,000 people during week 1 of data collection and the second highest infection rate during week 2. Additionally, Ireland had the largest speed and jerk and the second largest acceleration during week 1, thereby increasing concern for a potential future outbreak in the country. However, all of these dynamic metrics decreased dramatically during week 2 (January 11-17, 2021), with acceleration and jerk actually transitioning to negative values, supporting the idea that Ireland’s mitigation strategy is proving to be effective, at least during the time period in question.

Portugal was also at high risk of increased transmission, with a transmission rate per 100,000 people in the top 5 countries of the region during both weeks. With respect to novel dynamic metrics, Portugal had the largest positive acceleration in the region during week 1 and the third largest in week 2. Additionally, the country’s jerk increased from a negative value to a positive value and the 7-day persistence effect almost doubled from week to week. This finding indicates that Portugal should consider implementing new policies to reduce transmission and specifically to restrict the evolution of superspreader events, given the increase in 7-day persistence and the fact that Portugal had the second highest death rate per 100,000 people in the region during week 2 (January 11-17, 2021). Residents of Portugal were not only highly likely to contract COVID-19 during this time period, but they were also more likely to die of the disease than residents of most other European countries.

Although some European countries showed signs of uncontrolled growth for the near future, many demonstrate decreasing dynamic metrics that provide reassurance that transmission is being controlled appropriately. However, based on these results, countries with increasing dynamic metrics that are most at risk of outbreaks include Andorra, Portugal, and Spain. Fortunately, Andorra’s population is relatively small for the region, potentially insulating regional policy makers and agencies from an overwhelming surge in COVID-19 cases. In contrast, Spain and Portugal are relatively large countries. Their caseloads and positive dynamic metrics suggest that these two countries would require substantial effort to control the COVID-19 spread. Regional coordination would be essential given the size of these countries from a population and economic perspective. Additionally, some countries such as the Czech Republic have very high 7-day persistence effects but decreasing speed and acceleration, indicating that the overall transmission in the country may be decreasing, but focused policy targeted toward preventing superspreader events may be helpful.

Europe experienced a surge in COVID-19 transmission due to the second wave of the pandemic [11,63-65]. Because infection rates had significantly increased across Europe, many governments imposed strict lockdowns shutting down European economies again. Since SARS-CoV-2 cases were first reported in Europe earlier in 2020, COVID-19–related research has kept pace and, consequently, fewer deaths have been reported [61]. The virus is still just as contagious and deadly, but targeted therapies have resulted in attenuation of death rates across countries [61].

### Limitations

Data are limited by granularity and collection method. Data were collected at the country level, which precludes local analysis of surveillance trends. Moreover, data collection mechanisms differ by country and may even differ by region within a given country. These different methods lead to weekend effects, missing data points, and other contamination.

### Comparison With Prior Work

This study is part of a broader research program at Northwestern Feinberg School of Medicine, The Global SARS-CoV-2 Surveillance Project: Policy, Persistence, & Transmission. This research program developed novel surveillance metrics to include speed, acceleration, jerk, and 7-day persistence at the country level [17,66]. We have also derived surveillance metrics for all global regions.

### Conclusion

Static and dynamic public health surveillance tools provide a more complete picture of the progression of the COVID-19 pandemic across countries and regions. Although static measures, including infection rates and death rates, capture data at a given point in time, they are less successful in assessing population health over a period of weeks or months. By including speed, acceleration, jerk, and 7-day persistence, public health officials may design policies with an eye to the future. According to surveillance data, all countries in Europe that were at the highest risk during the second wave of the COVID-19 pandemic shared a number of characteristics. The United Kingdom, Spain, and Portugal demonstrated high infection rates, jerk, and 7-day persistence rates. Looking ahead, policy makers in these countries and the region at large should be concerned about growth in the already substantial number of COVID-19 cases over the short term. Given the substantial 7-day persistence rates in large countries such as the United Kingdom, Spain, and the Czech Republic, it is imperative that efforts be made to target superspreader events. Analysis of subsequent surveillance data using both static and dynamic tools can help confirm the efficaciousness of new policies.

## Appendix

Multimedia Appendix 1 Tables S1-S10. Static and novel surveillance metrics for specific weeks.

Multimedia Appendix 2 Map showing global weekly explosive growth potential of COVID-19 cases.

Multimedia Appendix 3 Map showing weekly speed of virus transmission by country in Europe.

Multimedia Appendix 4 Map showing weekly acceleration/jerk by country in Europe.

## Abbreviations

EU: European Union
GMM: generalized method of moments
WB: World Bank
WHO: World Health Organization
