# Supplementary material for: Surveillance of the Second Wave of COVID-19 in Europe: Longitudinal Trend Analyses
Source: JMIR Public Health Surveill. 2021 Apr 28;7(4):e25695. doi: 10.2196/25695 (PMC8080962; doi:10.2196/25695)
Supplement: Multimedia Appendix 1 [file publichealth_v7i4e25695_app1.docx]

**Table S1. Static Surveillance Metrics for the Week of 11/30-12/06**

| **Country** | **New Weekly COVID Cases** | **Cumulative COVID Cases** | **7 Day Moving Average New Cases** | **Infection Rate per 100K** | **New Weekly Deaths** | **Cumulative Deaths** | **7 Day Moving Average of Death** | **Death Rate per 100K** |
| --- | --- | --- | --- | --- | --- | --- | --- | --- |
| Albania | 840 | 42988 | 766.14 | 29.19 | 16 | 905 | 15.29 | 0.56 |
| Andorra | 45 | 7050 | 48.29 | 58.24 | 0 | 78 | 0.29 | 0 |
| Austria | 2741 | 303430 | 3388.86 | 30.43 | 83 | 3840 | 105 | 0.92 |
| Belarus | 1878 | 147157 | 1735.57 | 19.87 | 9 | 1207 | 8 | 0.10 |
| Belgium | 1814 | 591756 | 2165.29 | 15.65 | 66 | 17320 | 110.43 | 0.57 |
| Bosnia & Herzegovina | 739 | 94507 | 1019 | 22.52 | 30 | 2952 | 47.43 | 0.91 |
| Bulgaria | 577 | 161421 | 2705 | 8.30 | 68 | 4797 | 140.43 | 0.98 |
| Croatia | 2899 | 150353 | 3391.57 | 70.62 | 72 | 2174 | 66 | 1.75 |
| Czech Republic | 2654 | 546833 | 3872.86 | 24.78 | 87 | 8902 | 109.14 | 0.81 |
| Denmark | 1749 | 91131 | 1608.43 | 30.20 | 7 | 885 | 8 | 0.12 |
| Estonia | 478 | 14978 | 418 | 36.03 | 5 | 131 | 2.71 | 0.38 |
| Finland | 413 | 27631 | 428.86 | 7.45 | 0 | 415 | 3.14 | 0 |
| France | 10949 | 2347595 | 10725 | 16.77 | 174 | 55247 | 405.29 | 0.27 |
| Germany | 10910 | 1194550 | 19837 | 13.02 | 150 | 18989 | 383.29 | 0.18 |
| Greece | 903 | 115471 | 1606.29 | 8.66 | 101 | 3003 | 97.43 | 0.97 |
| Hungary | 6697 | 250278 | 5535.86 | 69.32 | 162 | 5868 | 170.86 | 1.68 |
| Iceland | 6 | 5482 | 14.43 | 1.76 | 0 | 33 | 0.14 | 0 |
| Ireland | 298 | 74246 | 286.43 | 6.04 | 0 | 2099 | 6.71 | 0 |
| Italy | 18887 | 1728878 | 20528.57 | 31.24 | 564 | 60078 | 739.14 | 0.93 |
| Latvia | 526 | 21313 | 619.71 | 27.89 | 9 | 262 | 9.29 | 0.48 |
| Lithuania | 2219 | 74649 | 2065.14 | 81.51 | 15 | 626 | 19 | 0.55 |
| Luxembourg | 0 | 37017 | 515.43 | 0 | 0 | 345 | 6.43 | 0 |
| Malta | 97 | 10520 | 109.71 | 21.97 | 2 | 151 | 2.57 | 0.45 |
| Moldova | 808 | 116365 | 1335.43 | 20.03 | 16 | 2419 | 18.43 | 0.40 |
| Netherlands | 6912 | 566628 | 5613.14 | 40.34 | 25 | 9768 | 48.86 | 0.15 |
| Norway | 250 | 38323 | 356.43 | 4.61 | 0 | 354 | 3.71 | 0 |
| Poland | 9176 | 1063449 | 11196.29 | 24.25 | 228 | 20089 | 437.14 | 0.60 |
| Portugal | 3834 | 322474 | 3953.57 | 37.60 | 87 | 4963 | 76.57 | 0.85 |
| Romania | 5231 | 513576 | 6005.71 | 27.19 | 134 | 12320 | 16 | 0.70 |
| San Marino | 0 | 1789 | 29 | 0 | 0 | 48 | 0.43 | 0 |
| Serbia | 5809 | 219652 | 7205.43 | 66.48 | 58 | 1949 | 57.14 | 0.66 |
| Slovakia | 1269 | 116731 | 1571.14 | 23.24 | 15 | 997 | 25.71 | 0.27 |
| Slovenia | 1030 | 85805 | 1489.14 | 49.54 | 44 | 1744 | 51.43 | 2.12 |
| Spain | 0 | 1684647 | 8062.71 | 0 | 0 | 46252 | 226.29 | 0 |
| Sweden | 0 | 278912 | 5111.86 | 0 | 0 | 7067 | 55.14 | 0 |
| Switzerland | 0 | 344497 | 3743.86 | 0 | 25 | 5349 | 99.86 | 0.29 |
| Ukraine | 11928 | 834913 | 13258.29 | 27.27 | 177 | 14054 | 205.86 | 0.40 |
| United Kingdom | 17372 | 1727751 | 15206.57 | 25.59 | 231 | 61342 | 428.57 | 0.34 |
| **Region** | 131938 | 15954746 | 149725.86 | 22.04 | 2660 | 379022 | 4352.14 | 0.44 |

**Table S2. Static Surveillance Metrics for the Week of 12/07-12/13**

| **Country** | **New Weekly COVID Cases** | **Cumulative COVID Cases** | **7 Day Moving Average New Cases** | **Infection Rate per 100K** | **New Weekly Deaths** | **Cumulative Deaths** | **7 Day Moving Average of Death** | **Death Rate per 100K** |
| --- | --- | --- | --- | --- | --- | --- | --- | --- |
| Albania | 788 | 48530 | 791.71 | 27.38 | 14 | 1003 | 14 | 0.49 |
| Andorra | 50 | 7338 | 41.14 | 64.71 | 1 | 79 | 0.14 | 1.29 |
| Austria | 2641 | 322463 | 2719.00 | 29.32 | 58 | 4473 | 90.43 | 0.64 |
| Belarus | 1961 | 160295 | 1876.86 | 20.75 | 9 | 1263 | 8 | 0.10 |
| Belgium | 4978 | 608137 | 2340.14 | 42.95 | 159 | 17951 | 90.14 | 1.37 |
| Bosnia & Herzegovina | 811 | 101117 | 944.29 | 24.72 | 38 | 3336 | 54.86 | 1.16 |
| Bulgaria | 497 | 179449 | 2575.43 | 7.15 | 62 | 5688 | 127.29 | 0.89 |
| Croatia | 3363 | 175886 | 3647.57 | 81.92 | 78 | 2640 | 66.57 | 1.90 |
| Czech Republic | 3657 | 579079 | 4606.57 | 34.15 | 85 | 9535 | 90.43 | 0.79 |
| Denmark | 2645 | 110305 | 2739.14 | 45.66 | 6 | 941 | 8.00 | 0.10 |
| Estonia | 342 | 18055 | 439.57 | 25.78 | 1 | 149 | 2.57 | 0.08 |
| Finland | 360 | 30810 | 454.14 | 6.50 | 0 | 453 | 5.43 | 0 |
| France | 25480 | 2432559 | 12137.71 | 39.04 | 344 | 58015 | 395.43 | 0.53 |
| Germany | 14709 | 1350810 | 22322.86 | 17.56 | 206 | 22106 | 445.29 | 0.25 |
| Greece | 692 | 124534 | 1294.71 | 6.64 | 85 | 3625 | 88.86 | 0.82 |
| Hungary | 4153 | 280400 | 4303.14 | 42.99 | 181 | 6965 | 156.71 | 1.87 |
| Iceland | 5 | 5557 | 10.71 | 1.47 | 0 | 34 | 0.14 | 0 |
| Ireland | 429 | 76185 | 277.00 | 8.69 | 1 | 2124 | 3.57 | 0.02 |
| Italy | 17937 | 1843712 | 16404.86 | 29.67 | 484 | 64520 | 634.57 | 0.80 |
| Latvia | 629 | 25675 | 623.14 | 33.35 | 25 | 349 | 12.43 | 1.33 |
| Lithuania | 2849 | 93101 | 2636 | 104.65 | 24 | 815 | 27 | 0.88 |
| Luxembourg | 0 | 40755 | 534 | 0 | 0 | 392 | 6.71 | 0 |
| Malta | 52 | 11153 | 90.43 | 11.78 | 3 | 169 | 2.57 | 0.68 |
| Moldova | 795 | 126518 | 1450.43 | 19.71 | 25 | 2572 | 21.86 | 0.62 |
| Netherlands | 9937 | 623567 | 8134.14 | 57.99 | 30 | 10134 | 52.29 | 0.18 |
| Norway | 200 | 41003 | 382.86 | 3.69 | 0 | 387 | 4.71 | 0 |
| Poland | 8976 | 1135676 | 10318.14 | 23.72 | 188 | 22864 | 396.43 | 0.50 |
| Portugal | 4044 | 348744 | 3752.86 | 39.66 | 98 | 5559 | 85.14 | 0.96 |
| Romania | 4435 | 556335 | 6108.43 | 23.05 | 121 | 13385 | 152.14 | 0.63 |
| San Marino | 0 | 1932 | 20.43 | 0 | 0 | 51 | 0.43 | 0 |
| Serbia | 4995 | 266432 | 6682.86 | 57.17 | 56 | 2331 | 54.57 | 0.64 |
| Slovakia | 2190 | 132984 | 2321.86 | 40.11 | 27 | 1176 | 25.57 | 0.49 |
| Slovenia | 833 | 96314 | 1501.29 | 40.07 | 22 | 2063 | 45.57 | 1.06 |
| Spain | 0 | 1730575 | 6561.14 | 0 | 0 | 47624 | 196 | 0 |
| Sweden | 0 | 320098 | 5883.71 | 0 | 0 | 7514 | 63.86 | 0 |
| Switzerland | 0 | 373831 | 4190.57 | 0 | 9 | 5985 | 90.86 | 0.10 |
| Ukraine | 9605 | 918444 | 11933 | 21.96 | 163 | 15691 | 233.86 | 0.37 |
| United Kingdom | 18541 | 1854490 | 18105.57 | 27.31 | 144 | 64267 | 417.86 | 0.21 |
| **Region** | 153579 | 17152848 | 155763.14 | 25.66 | 2747 | 408228 | 4172.29 | 0.46 |

**Table S3. Static Surveillance Metrics for the Week of 12/14-12/20**

| **Country** | **New Weekly COVID Cases** | **Cumulative COVID Cases** | **7 Day Moving Average New Cases** | **Infection Rate per 100K** | **New Weekly Deaths** | **Cumulative Deaths** | **7 Day Moving Average of Death** | **Death Rate per 100K** |
| --- | --- | --- | --- | --- | --- | --- | --- | --- |
| Albania | 461 | 53003 | 639 | 16.02 | 14 | 1088 | 12.14 | 0.49 |
| Andorra | 17 | 7577 | 34.14 | 22 | 1 | 81 | 0.29 | 1.29 |
| Austria | 1645 | 338854 | 2341.57 | 18.26 | 142 | 5351 | 125.43 | 1.58 |
| Belarus | 1944 | 173523 | 1889.71 | 20.57 | 8 | 1324 | 8.71 | 0.08 |
| Belgium | 2170 | 625930 | 2541.86 | 18.72 | 81 | 18626 | 96.43 | 0.70 |
| Bosnia & Herzegovina | 0 | 105524 | 629.57 | 0 | 0 | 3625 | 41.29 | 0 |
| Bulgaria | 166 | 191195 | 1678 | 2.39 | 58 | 6609 | 131.57 | 0.83 |
| Croatia | 1975 | 194962 | 2725.14 | 48.11 | 76 | 3177 | 76.71 | 1.85 |
| Czech Republic | 5304 | 624140 | 6437.29 | 49.53 | 60 | 10331 | 113.71 | 0.56 |
| Denmark | 2835 | 134999 | 3527.71 | 48.95 | 16 | 1035 | 13.43 | 0.28 |
| Estonia | 575 | 21794 | 534.14 | 43.35 | 4 | 174 | 3.57 | 0.30 |
| Finland | 309 | 33162 | 336 | 5.58 | 0 | 489 | 5.14 | 0 |
| France | 12799 | 2529756 | 13885.29 | 19.61 | 131 | 60665 | 378.57 | 0.20 |
| Germany | 6444 | 1514962 | 23450.29 | 7.69 | 229 | 26400 | 613.43 | 0.27 |
| Greece | 587 | 131072 | 934 | 5.63 | 70 | 4172 | 78.14 | 0.67 |
| Hungary | 2967 | 302989 | 3227 | 30.71 | 185 | 8099 | 162 | 1.92 |
| Iceland | 21 | 5642 | 12.14 | 6.15 | 0 | 34 | 0 | 0 |
| Ireland | 766 | 79542 | 479.57 | 15.51 | 4 | 2158 | 4.86 | 0.08 |
| Italy | 15102 | 1953185 | 15639 | 24.98 | 352 | 68799 | 611.29 | 0.58 |
| Latvia | 643 | 30940 | 752.14 | 34.09 | 12 | 439 | 12.86 | 0.64 |
| Lithuania | 2930 | 112359 | 2751.14 | 107.63 | 14 | 1019 | 29.14 | 0.51 |
| Luxembourg | 0 | 43728 | 424.71 | 0 | 0 | 434 | 6 | 0 |
| Malta | 93 | 11714 | 80.14 | 21.06 | 3 | 190 | 3 | 0.68 |
| Moldova | 629 | 135207 | 1241.29 | 15.59 | 25 | 2752 | 25.71 | 0.62 |
| Netherlands | 13072 | 700322 | 10965 | 76.29 | 32 | 10578 | 63.43 | 0.19 |
| Norway | 323 | 43905 | 414.57 | 5.96 | 0 | 404 | 2.43 | 0 |
| Poland | 8590 | 1202700 | 9574.86 | 22.70 | 143 | 25397 | 361.86 | 0.38 |
| Portugal | 3334 | 374121 | 3625.29 | 32.70 | 71 | 6134 | 82.14 | 0.70 |
| Romania | 3350 | 591294 | 4994.14 | 17.41 | 98 | 14394 | 144.14 | 0.51 |
| San Marino | 0 | 2107 | 25 | 0 | 0 | 55 | 0.57 | 0 |
| Serbia | 3534 | 300062 | 4804.29 | 40.45 | 54 | 2686 | 50.71 | 0.62 |
| Slovakia | 2061 | 151336 | 2621.71 | 37.75 | 45 | 1556 | 54.29 | 0.82 |
| Slovenia | 886 | 105899 | 1369.29 | 42.62 | 39 | 2353 | 41.43 | 1.88 |
| Spain | 0 | 1797236 | 9523 | 0 | 0 | 48926 | 186 | 0 |
| Sweden | 0 | 367120 | 6717.43 | 0 | 0 | 7993 | 68.43 | 0 |
| Switzerland | 0 | 403989 | 4308.29 | 0 | 20 | 6622 | 91 | 0.23 |
| Ukraine | 8763 | 991700 | 10465.14 | 20.04 | 129 | 17201 | 215.71 | 0.29 |
| United Kingdom | 36084 | 2046161 | 27381.57 | 53.15 | 326 | 67503 | 462.29 | 0.48 |
| **Region** | 140379 | 18433711 | 167233.29 | 23.45 | 2442 | 438873 | 4377.86 | 0.41 |

**Table S4. Static Surveillance Metrics for the Week of 12/21-12/27**

| **Country** | **New Weekly COVID Cases** | **Cumulative COVID Cases** | **7 Day Moving Average New Cases** | **Infection Rate per 100K** | **New Weekly Deaths** | **Cumulative Deaths** | **7 Day Moving Average of Death** | **Death Rate per 100K** |
| --- | --- | --- | --- | --- | --- | --- | --- | --- |
| Albania | 499 | 56254 | 464.43 | 17.34 | 10 | 1153 | 9.29 | 0.35 |
| Andorra | 15 | 7821 | 34.86 | 19.41 | 0 | 83 | 0.29 | 0 |
| Austria | 1408 | 351892 | 1862.57 | 15.63 | 38 | 5881 | 75.71 | 0.42 |
| Belarus | 1825 | 186747 | 1889.14 | 19.31 | 9 | 1385 | 8.71 | 0.10 |
| Belgium | 847 | 638877 | 1849.57 | 7.31 | 42 | 19200 | 82.00 | 0.36 |
| Bosnia & Herzegovina | 361 | 109691 | 595.29 | 11 | 30 | 3953 | 46.86 | 0.91 |
| Bulgaria | 332 | 197716 | 931.57 | 4.78 | 41 | 7164 | 79.29 | 0.59 |
| Croatia | 618 | 204930 | 1424 | 15.05 | 58 | 3671 | 70.57 | 1.41 |
| Czech Republic | 3030 | 670599 | 6637 | 28.29 | 94 | 11044 | 101.86 | 0.88 |
| Denmark | 2188 | 153952 | 2707.57 | 37.77 | 21 | 1174 | 19.86 | 0.36 |
| Estonia | 365 | 25392 | 514.00 | 27.52 | 0 | 204 | 4.29 | 0 |
| Finland | 156 | 34977 | 259.29 | 2.82 | 0 | 524 | 5 | 0 |
| France | 8822 | 2616510 | 12393.43 | 13.52 | 173 | 62867 | 314.57 | 0.27 |
| Germany | 12399 | 1658639 | 20525.29 | 14.80 | 351 | 30297 | 556.71 | 0.42 |
| Greece | 342 | 135456 | 626.29 | 3.28 | 53 | 4606 | 62.00 | 0.51 |
| Hungary | 698 | 316060 | 1867.29 | 7.23 | 96 | 9047 | 135.43 | 0.99 |
| Iceland | 0 | 5683 | 5.86 | 0 | 0 | 34 | 0 | 0 |
| Ireland | 735 | 86129 | 941.00 | 14.89 | 4 | 2204 | 6.57 | 0.08 |
| Italy | 8937 | 2047696 | 13501.57 | 14.78 | 305 | 71925 | 446.57 | 0.50 |
| Latvia | 511 | 36330 | 770 | 27.09 | 44 | 559 | 17.14 | 2.33 |
| Lithuania | 1667 | 130598 | 2605.57 | 61.24 | 26 | 1254 | 33.57 | 0.96 |
| Luxembourg | 0 | 45209 | 211.57 | 0 | 0 | 458 | 3.43 | 0 |
| Malta | 84 | 12325 | 87.29 | 19.02 | 4 | 210 | 2.86 | 0.91 |
| Moldova | 359 | 141355 | 878.29 | 8.90 | 8 | 2891 | 19.86 | 0.20 |
| Netherlands | 9102 | 774009 | 10526.71 | 53.12 | 28 | 11090 | 73.14 | 0.16 |
| Norway | 381 | 47059 | 450.57 | 7.03 | 0 | 421 | 2.43 | 0 |
| Poland | 3842 | 1257799 | 7871.29 | 10.15 | 57 | 27118 | 245.86 | 0.15 |
| Portugal | 1577 | 394573 | 2921.71 | 15.47 | 63 | 6619 | 69.29 | 0.62 |
| Romania | 2049 | 615809 | 3502.14 | 10.65 | 122 | 15230 | 119.43 | 0.63 |
| San Marino | 0 | 2231 | 17.71 | 0 | 0 | 56 | 0.14 | 0 |
| Serbia | 2693 | 326060 | 3714 | 30.82 | 47 | 3030 | 49.14 | 0.54 |
| Slovakia | 874 | 167523 | 2312.43 | 16.01 | 41 | 1774 | 31.14 | 0.75 |
| Slovenia | 622 | 114806 | 1272.43 | 29.92 | 33 | 2565 | 30.29 | 1.59 |
| Spain | 0 | 1854951 | 8245.00 | 0 | 0 | 49824 | 128.29 | 0 |
| Sweden | 0 | 396048 | 4132.57 | 0 | 0 | 8279 | 40.86 | 0 |
| Switzerland | 0 | 428197 | 3458.29 | 0 | 17 | 7210 | 84.00 | 0.20 |
| Ukraine | 6548 | 1056265 | 9223.57 | 14.97 | 83 | 18472 | 181.57 | 0.19 |
| United Kingdom | 32493 | 2295228 | 35581.00 | 47.86 | 347 | 70860 | 479.57 | 0.51 |
| **Region** | 106379 | 19601396 | 149161.00 | 17.77 | 2245 | 464336 | 3637.57 | 0.38 |

**Table S5. Static Surveillance Metrics for the Week of 12/28-01/03**

| **Country** | **New Weekly COVID Cases** | **Cumulative COVID Cases** | **7 Day Moving Average New Cases** | **Infection Rate per 100K** | **New Weekly Deaths** | **Cumulative Deaths** | **7 Day Moving Average of Death** | **Death Rate per 100K** |
| --- | --- | --- | --- | --- | --- | --- | --- | --- |
| Albania | 447 | 59438 | 454.86 | 15.53 | 3 | 1193 | 5.71 | 0.10 |
| Andorra | 26 | 8192 | 53 | 33.65 | 0 | 84 | 0.14 | 0 |
| Austria | 1466 | 365768 | 1982.29 | 16.28 | 49 | 6324 | 63.29 | 0.54 |
| Belarus | 1837 | 199962 | 1887.86 | 19.44 | 9 | 1451 | 9.43 | 0.10 |
| Belgium | 842 | 650011 | 1590.57 | 7.27 | 57 | 19701 | 71.57 | 0.49 |
| Bosnia & Herzegovina | 502 | 112645 | 422 | 15.30 | 45 | 4131 | 25.43 | 1.37 |
| Bulgaria | 171 | 203051 | 762.14 | 2.46 | 34 | 7678 | 73.43 | 0.49 |
| Croatia | 696 | 212958 | 1146.86 | 16.95 | 56 | 4072 | 57.29 | 1.36 |
| Czech Republic | 5012 | 740481 | 9983.14 | 46.80 | 133 | 11960 | 130.86 | 1.24 |
| Denmark | 1176 | 169358 | 2200.86 | 20.30 | 29 | 1374 | 28.57 | 0.50 |
| Estonia | 342 | 29131 | 534.14 | 25.78 | 3 | 244 | 5.71 | 0.23 |
| Finland | 168 | 36772 | 256.43 | 3.03 | 0 | 561 | 5.29 | 0 |
| France | 12495 | 2712975 | 13780.71 | 19.14 | 116 | 65164 | 328.14 | 0.18 |
| Germany | 10356 | 1783896 | 17893.86 | 12.36 | 311 | 34791 | 642 | 0.37 |
| Greece | 390 | 140099 | 663.29 | 3.74 | 36 | 4957 | 50.14 | 0.35 |
| Hungary | 1307 | 327995 | 1705 | 13.53 | 103 | 9884 | 119.57 | 1.07 |
| Iceland | 0 | 5754 | 10.14 | 0 | 0 | 35 | 0.14 | 0 |
| Ireland | 4961 | 101887 | 2251.14 | 100.47 | 7 | 2259 | 7.86 | 0.14 |
| Italy | 14245 | 2155446 | 15392.86 | 23.56 | 347 | 75332 | 486.71 | 0.57 |
| Latvia | 568 | 42497 | 881 | 30.11 | 12 | 680 | 17.29 | 0.64 |
| Lithuania | 1238 | 146637 | 2291.29 | 45.48 | 29 | 1643 | 55.57 | 1.07 |
| Luxembourg | 0 | 46415 | 172.29 | 0 | 0 | 495 | 5.29 | 0 |
| Malta | 85 | 13082 | 108.14 | 19.25 | 0 | 220 | 1.43 | 0 |
| Moldova | 179 | 145873 | 645.43 | 4.44 | 17 | 3037 | 20.86 | 0.42 |
| Netherlands | 7453 | 832702 | 8384.71 | 43.50 | 47 | 11707 | 88.14 | 0.27 |
| Norway | 450 | 50716 | 522.43 | 8.30 | 0 | 436 | 2.14 | 0 |
| Poland | 5782 | 1318562 | 8680.43 | 15.28 | 61 | 29119 | 285.86 | 0.16 |
| Portugal | 3384 | 427254 | 4668.71 | 33.19 | 73 | 7118 | 71.29 | 0.72 |
| Romania | 3034 | 640429 | 3517.14 | 15.77 | 60 | 15979 | 107 | 0.31 |
| San Marino | 0 | 2428 | 28.14 | 0 | 0 | 59 | 0.43 | 0 |
| Serbia | 1966 | 343870 | 2544.29 | 22.50 | 37 | 3325 | 42.14 | 0.42 |
| Slovakia | 1219 | 187463 | 2848.57 | 22.33 | 67 | 2318 | 77.71 | 1.23 |
| Slovenia | 743 | 125086 | 1468.57 | 35.74 | 29 | 2803 | 34 | 1.39 |
| Spain | 0 | 1928265 | 10473.43 | 0 | 0 | 50837 | 144.71 | 0 |
| Sweden | 0 | 437379 | 5904.43 | 0 | 0 | 8727 | 64 | 0 |
| Switzerland | 0 | 452296 | 3442.71 | 0 | 18 | 7747 | 76.71 | 0.21 |
| Ukraine | 4881 | 1107137 | 7267.43 | 11.16 | 132 | 19630 | 165.43 | 0.30 |
| United Kingdom | 55157 | 2662699 | 52495.86 | 81.25 | 455 | 75137 | 611 | 0.67 |
| **Region** | 142578 | 20926609 | 174678.29 | 23.82 | 2375 | 492212 | 3982.29 | 0.40 |

**Table S6. Novel Surveillance Metrics for the Week of 11/30-12/06**

| **Country** | **SPEED: Daily positives per 100K (weekly average of new daily cases per 100K)** | **ACCELERATION: day-to-day change in the number of positives per day, weekly average, per 100K** | **JERK: week over week change in ACCELERATION, per 100K** | **7-DAY PERSISTENCE EFFECT on SPEED**  **(# new cases per day per 100Kattributed to new cases 7 days ago)** |
| --- | --- | --- | --- | --- |
| Albania | 26.62 | 0.02 | -1.47 | 21.63 |
| Andorra | 62.49 | -10.54 | -19.78 | 75.53 |
| Austria | 37.63 | -2.07 | -0.13 | 46.21 |
| Belarus | 18.37 | 0.29 | -0.02 | 14.91 |
| Belgium | 18.68 | -0.42 | 0.96 | 19.68 |
| Bosnia & Herzegovina | 31.06 | 0.33 | 1.65 | 28.74 |
| Bulgaria | 38.93 | -0.33 | -0.84 | 38.06 |
| Croatia | 82.62 | -0.07 | -0.41 | 71.37 |
| Czech Republic | 36.16 | 2.11 | 3.30 | 32.82 |
| Denmark | 27.77 | 1.85 | 0.67 | 19.60 |
| Estonia | 31.51 | 1.34 | -0.67 | 22.46 |
| Finland | 7.74 | 0.23 | 0.44 | 6.91 |
| France | 16.43 | 0.26 | 0.17 | 15.64 |
| Germany | 23.68 | 1.32 | -0.78 | 18.84 |
| Greece | 15.41 | -0.40 | 0.10 | 15.48 |
| Hungary | 57.30 | -0.18 | 0.92 | 48.90 |
| Iceland | 4.23 | -0.17 | 0.29 | 3.90 |
| Ireland | 5.80 | 0 | -0.62 | 4.61 |
| Italy | 33.95 | -0.42 | 0.83 | 37.32 |
| Latvia | 32.86 | 0.85 | 0.54 | 26.16 |
| Lithuania | 75.86 | 1.25 | -2.59 | 61.80 |
| Luxembourg | 82.34 | 0 | 0 | 74.56 |
| Malta | 24.85 | -1.49 | -1.33 | 24 |
| Moldova | 33.10 | -1.26 | -1.32 | 28.79 |
| Netherlands | 32.76 | 1.01 | -0.72 | 26.17 |
| Norway | 6.57 | -0.08 | 0.13 | 7.23 |
| Poland | 29.58 | -0.87 | 0.17 | 41.85 |
| Portugal | 38.77 | -0.36 | -2.07 | 42.73 |
| Romania | 31.22 | -0.24 | -0.19 | 35.19 |
| San Marino | 85.47 | 0 | -0.42 | 72.04 |
| Serbia | 82.47 | -0.60 | -0.38 | 70.45 |
| Slovakia | 28.78 | 0.44 | -0.98 | 22.25 |
| Slovenia | 71.63 | -0.65 | -1.05 | 62.01 |
| Spain | 17.24 | 0 | 0 | 19.57 |
| Sweden | 50.62 | 0 | 0 | 44.14 |
| Switzerland | 43.26 | 0 | 0 | 40.95 |
| Ukraine | 30.32 | -0.44 | 0.35 | 29.23 |
| United Kingdom | 22.40 | 1.10 | 1.16 | 19.89 |
| **Region** | 27.99 | 0.23 | 0.15 | 27.30 |

**Table S7. Novel Surveillance Metrics for the Week of 12/07-12/13**

| **Country** | **SPEED: Daily positives per 100K (weekly average of new daily cases per 100K)** | **ACCELERATION: day-to-day change in the number of positives per day, weekly average, per 100K** | **JERK: week over week change in ACCELERATION, per 100K** | **7-DAY PERSISTENCE EFFECT on SPEED**  **(# new cases per day per 100K attributed to new cases 7 days ago)** |
| --- | --- | --- | --- | --- |
| Albania | 27.51 | -0.26 | -0.42 | 23.85 |
| Andorra | 53.25 | 0.92 | 0.55 | 55.99 |
| Austria | 30.19 | -0.16 | 0.16 | 33.71 |
| Belarus | 19.86 | 0.13 | 0.01 | 16.45 |
| Belgium | 20.19 | 3.90 | 3.58 | 16.74 |
| Bosnia & Herzegovina | 28.78 | 0.31 | 1.24 | 27.83 |
| Bulgaria | 37.06 | -0.16 | 1.38 | 34.88 |
| Croatia | 88.85 | 1.61 | 1.44 | 74.01 |
| Czech Republic | 43.02 | 1.34 | -4.59 | 32.40 |
| Denmark | 47.29 | 2.21 | -1.34 | 24.88 |
| Estonia | 33.14 | -1.46 | -3.58 | 28.23 |
| Finland | 8.20 | -0.14 | 0.08 | 6.93 |
| France | 18.60 | 3.18 | 6.00 | 14.72 |
| Germany | 26.64 | 0.65 | 1.39 | 21.21 |
| Greece | 12.42 | -0.29 | -0.03 | 13.81 |
| Hungary | 44.54 | -3.76 | -3.06 | 51.34 |
| Iceland | 3.14 | -0.04 | 0 | 3.79 |
| Ireland | 5.61 | 0.38 | 0.98 | 5.20 |
| Italy | 27.13 | -0.22 | 0.05 | 30.42 |
| Latvia | 33.04 | 0.78 | 1.79 | 29.43 |
| Lithuania | 96.83 | 3.31 | 0.92 | 67.96 |
| Luxembourg | 85.31 | 0 | 0 | 73.77 |
| Malta | 20.48 | -1.46 | -2.04 | 22.26 |
| Moldova | 35.96 | -0.05 | 0.91 | 29.66 |
| Netherlands | 47.47 | 2.52 | 0.44 | 29.35 |
| Norway | 7.06 | -0.13 | -0.37 | 5.89 |
| Poland | 27.26 | -0.08 | 0.27 | 26.50 |
| Portugal | 36.80 | 0.29 | 2.64 | 34.74 |
| Romania | 31.75 | -0.59 | 0.70 | 27.97 |
| San Marino | 60.21 | 0 | 3.79 | 76.57 |
| Serbia | 76.49 | -1.33 | 0.67 | 73.88 |
| Slovakia | 42.53 | 2.41 | -1.87 | 25.78 |
| Slovenia | 72.21 | -1.35 | -2.05 | 64.17 |
| Spain | 14.03 | 0 | 0 | 15.45 |
| Sweden | 58.26 | 0 | 0 | 45.35 |
| Switzerland | 48.42 | 0 | 0 | 38.75 |
| Ukraine | 27.29 | -0.76 | -0.45 | 27.16 |
| United Kingdom | 26.67 | 0.25 | -1.01 | 20.07 |
| **Region** | 28.59 | 0.52 | 0.76 | 25.07 |

**Table S8. Novel Surveillance Metrics for the Week of 12/14-12/20**

| **Country** | **SPEED: Daily positives per 100K (weekly average of new daily cases per 100K)** | **ACCELERATION: day-to-day change in the number of positives per day, weekly average, per 100K** | **JERK: week over week change in ACCELERATION, per 100K** | **7-DAY PERSISTENCE EFFECT on SPEED**  **(# new cases per day per 100Kattributed to new cases 7 days ago)** |
| --- | --- | --- | --- | --- |
| Albania | 22.20 | -1.62 | 0.07 | 24.65 |
| Andorra | 44.19 | -6.10 | -4.07 | 47.70 |
| Austria | 26 | -1.58 | -0.08 | 27.05 |
| Belarus | 20 | -0.03 | 0.04 | 17.79 |
| Belgium | 21.93 | -3.46 | -3.41 | 18.09 |
| Bosnia & Herzegovina | 19.19 | -3.53 | -3.01 | 25.79 |
| Bulgaria | 24.15 | -0.68 | -0.09 | 33.21 |
| Croatia | 66.38 | -4.83 | -0.02 | 79.60 |
| Czech Republic | 60.11 | 2.20 | -1.29 | 38.54 |
| Denmark | 60.90 | 0.47 | 1.13 | 42.37 |
| Estonia | 40.27 | 2.51 | 4.62 | 29.69 |
| Finland | 6.06 | -0.13 | 0.14 | 7.34 |
| France | 21.27 | -2.78 | -6.59 | 16.66 |
| Germany | 27.99 | -1.41 | -1.39 | 23.87 |
| Greece | 8.96 | -0.14 | 0.26 | 11.13 |
| Hungary | 33.40 | -1.75 | -0.27 | 39.91 |
| Iceland | 3.56 | 0.67 | 0.50 | 2.81 |
| Ireland | 9.71 | 0.97 | 0.19 | 5.03 |
| Italy | 25.87 | -0.67 | 0.18 | 24.31 |
| Latvia | 39.88 | 0.11 | -1.48 | 29.60 |
| Lithuania | 101.06 | 0.43 | 0.87 | 86.75 |
| Luxembourg | 67.85 | 0 | 0 | 76.42 |
| Malta | 18.15 | 1.33 | 3.56 | 18.35 |
| Moldova | 30.77 | -0.59 | 0.27 | 32.21 |
| Netherlands | 63.99 | 2.61 | 0.03 | 42.53 |
| Norway | 7.65 | 0.32 | 0.30 | 6.33 |
| Poland | 25.30 | -0.15 | -0.05 | 24.42 |
| Portugal | 35.55 | -0.99 | -0.18 | 32.97 |
| Romania | 25.96 | -0.81 | 0.07 | 28.45 |
| San Marino | 73.68 | 0 | -4.63 | 53.94 |
| Serbia | 54.99 | -2.39 | 0.54 | 68.52 |
| Slovakia | 48.02 | -0.34 | 1.12 | 38.10 |
| Slovenia | 65.86 | 0.36 | 2.33 | 64.70 |
| Spain | 20.37 | 0 | 0 | 12.57 |
| Sweden | 66.51 | 0 | 0 | 52.19 |
| Switzerland | 49.78 | 0 | 0 | 43.38 |
| Ukraine | 23.93 | -0.28 | 0.06 | 24.44 |
| United Kingdom | 40.33 | 3.69 | 2.49 | 23.89 |
| **Region** | 30.57 | -0.32 | -0.66 | 25.62 |

**Table S9. Novel Surveillance Metrics for the Week of 12/21-12/27**

| **Country** | **SPEED: Daily positives per 100K (weekly average of new daily cases per 100K)** | **ACCELERATION: day-to-day change in the number of positives per day, weekly average, per 100K** | **JERK: week over week change in ACCELERATION, per 100K** | **7-DAY PERSISTENCE EFFECT on SPEED**  **(# new cases per day per 100K attributed to new cases 7 days ago)** |
| --- | --- | --- | --- | --- |
| Albania | 16.14 | 0.19 | 1 | 19.89 |
| Andorra | 45.11 | -0.37 | -2.03 | 39.59 |
| Austria | 20.68 | -0.38 | 1 | 23.29 |
| Belarus | 19.99 | -0.18 | -0.16 | 17.92 |
| Belgium | 15.96 | -1.63 | 0.76 | 19.65 |
| Bosnia & Herzegovina | 18.14 | 1.57 | 2.46 | 17.19 |
| Bulgaria | 13.41 | 0.34 | 1.44 | 21.63 |
| Croatia | 34.69 | -4.72 | 3.64 | 59.47 |
| Czech Republic | 61.98 | -3.03 | 5.14 | 53.85 |
| Denmark | 46.75 | -1.60 | 1.99 | 54.56 |
| Estonia | 38.75 | -2.26 | -1.01 | 36.07 |
| Finland | 4.68 | -0.39 | -0.14 | 5.43 |
| France | 18.99 | -0.87 | 2.27 | 19.06 |
| Germany | 24.50 | 1.02 | 2.41 | 25.07 |
| Greece | 6.01 | -0.34 | 0.54 | 8.03 |
| Hungary | 19.33 | -3.36 | 0.85 | 29.93 |
| Iceland | 1.72 | -0.88 | -0.17 | 3.19 |
| Ireland | 19.06 | -0.09 | -2.33 | 8.70 |
| Italy | 22.33 | -1.46 | -0.06 | 23.17 |
| Latvia | 40.82 | -1 | 2.57 | 35.72 |
| Lithuania | 95.71 | -6.63 | -2.07 | 90.54 |
| Luxembourg | 33.80 | 0 | 0 | 60.78 |
| Malta | 19.77 | -0.29 | -2.78 | 16.26 |
| Moldova | 21.77 | -0.96 | 0.72 | 27.57 |
| Netherlands | 61.43 | -3.31 | -1.30 | 57.33 |
| Norway | 8.31 | 0.15 | 0.01 | 6.85 |
| Poland | 20.80 | -1.79 | 0.61 | 22.67 |
| Portugal | 28.65 | -2.46 | 1.21 | 31.85 |
| Romania | 18.20 | -0.97 | 1.83 | 23.26 |
| San Marino | 52.21 | 0 | 14.74 | 66.01 |
| Serbia | 42.51 | -1.38 | -0.25 | 49.26 |
| Slovakia | 42.35 | -3.11 | 2.42 | 43.02 |
| Slovenia | 61.21 | -1.81 | 5.67 | 59.01 |
| Spain | 17.63 | 0 | 0 | 18.25 |
| Sweden | 40.92 | 0 | 0 | 59.59 |
| Switzerland | 39.96 | 0 | 0 | 44.60 |
| Ukraine | 21.09 | -0.72 | 0.60 | 21.44 |
| United Kingdom | 52.41 | -0.76 | -2.34 | 36.14 |
| **Region** | 27.87 | -0.81 | 0.68 | 27.39 |

**Table S10. Novel Surveillance Metrics for the Week of 12/28-01/03**

| **Country** | **SPEED: Daily positives per 100K (weekly average of new daily cases per 100K)** | **ACCELERATION: day-to-day change in the number of positives per day, weekly average, per 100K** | **JERK: week over week change in ACCELERATION, per 100K** | **7-DAY PERSISTENCE EFFECT on SPEED**  **(# new cases per day per 100K**  **attributed to new cases 7 days ago)** |
| --- | --- | --- | --- | --- |
| Albania | 15.81 | -0.26 | -1.75 | 13.72 |
| Andorra | 68.60 | 2.03 | 2.22 | 39.60 |
| Austria | 22.01 | 0.09 | 0.15 | 17.87 |
| Belarus | 19.98 | 0.02 | 0.04 | 17.09 |
| Belgium | 13.72 | -0.01 | -0.12 | 13.99 |
| Bosnia & Herzegovina | 12.86 | 0.61 | 2.53 | 15.79 |
| Bulgaria | 10.97 | -0.33 | -0.07 | 11.81 |
| Croatia | 27.94 | 0.27 | 0.60 | 30.44 |
| Czech Republic | 93.22 | 2.64 | 1.66 | 54.33 |
| Denmark | 38 | -2.50 | -1.95 | 40.28 |
| Estonia | 40.27 | -0.25 | 0.42 | 33.55 |
| Finland | 4.63 | 0.03 | -0.04 | 4.07 |
| France | 21.11 | 0.80 | 0.72 | 16.44 |
| Germany | 21.36 | -0.35 | 0.10 | 21.32 |
| Greece | 6.36 | 0.07 | 0.07 | 5.24 |
| Hungary | 17.65 | 0.90 | 0.59 | 17.01 |
| Iceland | 2.97 | 0 | 0 | 1.54 |
| Ireland | 45.59 | 12.23 | 6.16 | 16.44 |
| Italy | 25.46 | 1.25 | 0.92 | 19.38 |
| Latvia | 46.71 | 0.43 | 1.08 | 35.43 |
| Lithuania | 84.17 | -2.25 | 2.23 | 83.16 |
| Luxembourg | 27.52 | 0 | 0 | 30.28 |
| Malta | 24.49 | 0.03 | 1.36 | 16.91 |
| Moldova | 16 | -0.64 | 0.46 | 19.13 |
| Netherlands | 48.93 | -1.37 | -0.34 | 52.79 |
| Norway | 9.64 | 0.18 | 0.09 | 7.15 |
| Poland | 22.94 | 0.73 | -0.07 | 18.20 |
| Portugal | 45.79 | 2.53 | -0.31 | 25.02 |
| Romania | 18.28 | 0.73 | 0.87 | 15.86 |
| San Marino | 82.94 | 0 | 0 | 46.77 |
| Serbia | 29.12 | -1.19 | 0.93 | 36.78 |
| Slovakia | 52.18 | 0.90 | -0.92 | 37.27 |
| Slovenia | 70.64 | 0.83 | 0.67 | 53.57 |
| Spain | 22.40 | 0 | 0 | 15.80 |
| Sweden | 58.46 | 0 | 0 | 36.66 |
| Switzerland | 39.78 | 0 | 0 | 35.80 |
| Ukraine | 16.62 | -0.54 | 0.35 | 18.26 |
| United Kingdom | 77.33 | 4.77 | -0.08 | 44.93 |
| **Region** | 31.63 | 0.86 | 0.32 | 24.21 |
