# Supplementary figures and images for: Surveillance of the Second Wave of COVID-19 in Europe: Longitudinal Trend Analyses
Source: JMIR Public Health Surveill. 2021 Apr 28;7(4):e25695. doi: 10.2196/25695 (PMC8080962; doi:10.2196/25695)

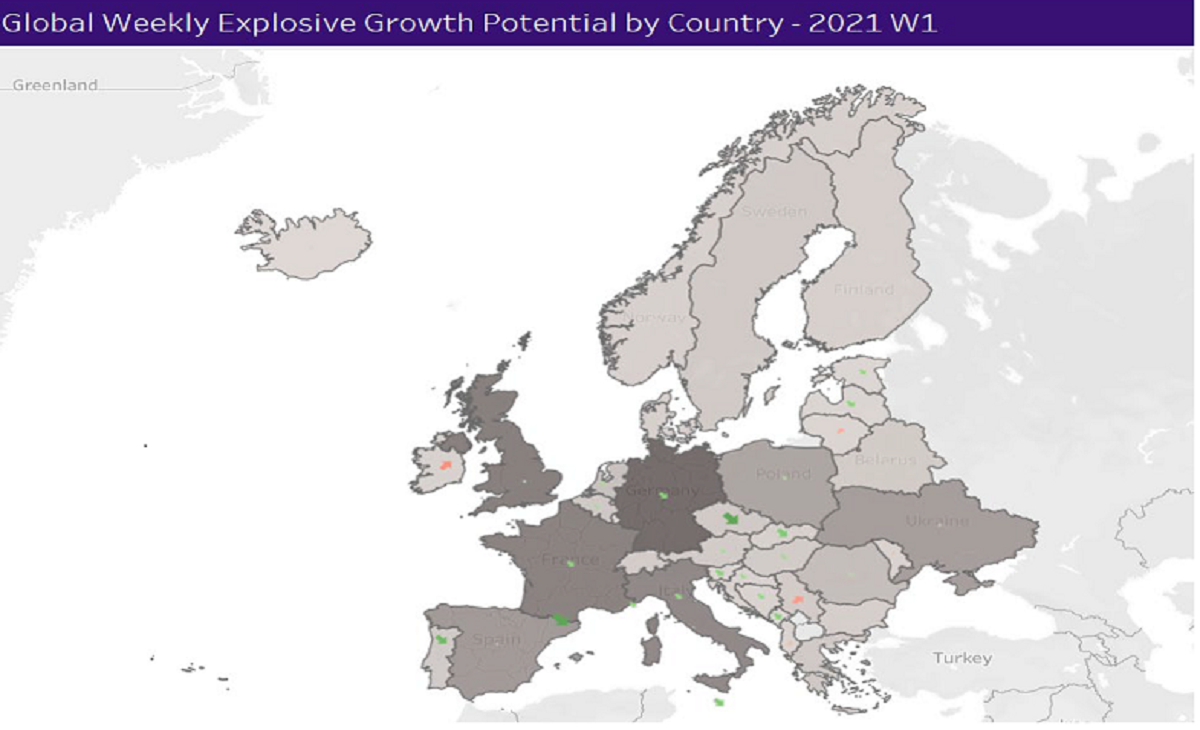

Supplement: Multimedia Appendix 2 [file publichealth_v7i4e25695_app2.png]

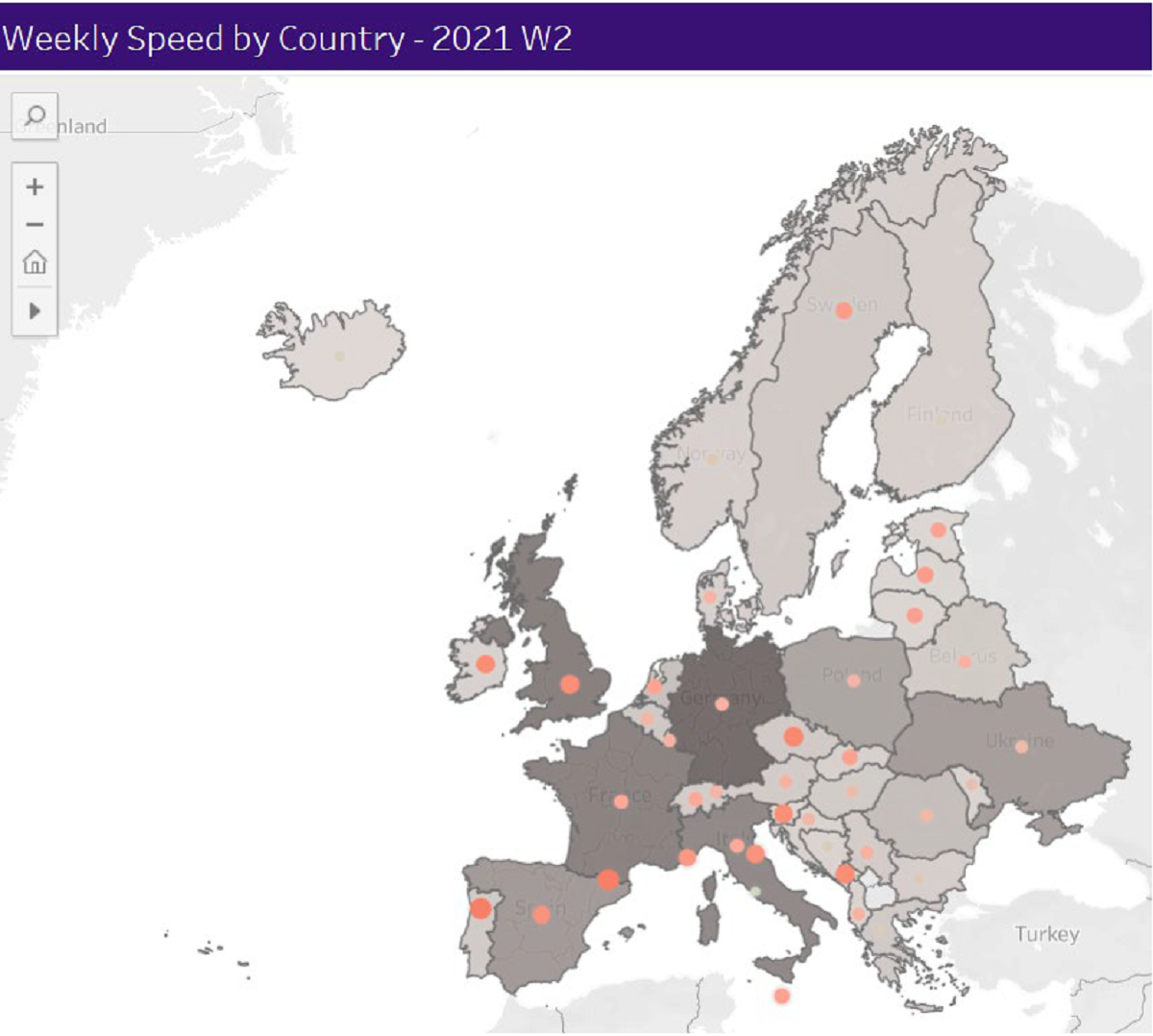

Supplement: Multimedia Appendix 3 [file publichealth_v7i4e25695_app3.png]

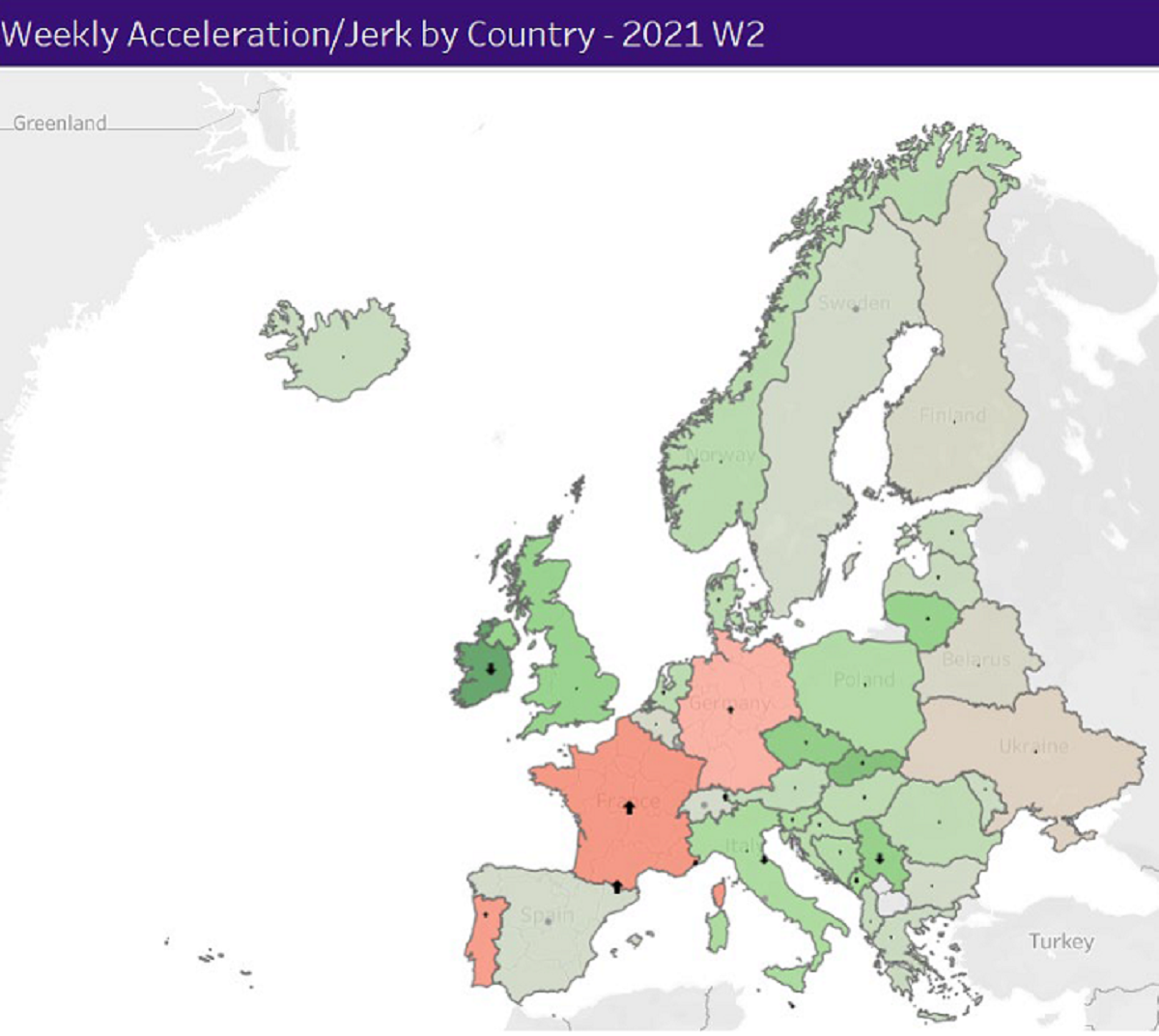

Supplement: Multimedia Appendix 4 [file publichealth_v7i4e25695_app4.png]
